# The importance of tissue specificity for RNA-seq: highlighting the errors of composite structure extractions

**DOI:** 10.1186/1471-2164-14-586

**Published:** 2013-08-28

**Authors:** Brian R Johnson, Joel Atallah, David C Plachetzki

**Affiliations:** 1 Department of Entomology, University of California, Davis, 1 Shields Ave, Davis, CA 95616, USA; 2 Department of Ecology and Evolution, University of California, Davis, 1 Shields Ave, Davis, CA 95616, USA

**Keywords:** RNA-seq, Tissue specificity, Genomics

## Abstract

**Background:**

A composite biological structure, such as an insect head or abdomen, contains many internal structures with distinct functions. Composite structures are often used in RNA-seq studies, though it is unclear how expression of the same gene in different tissues and structures within the same structure affects the measurement (or even utility) of the resulting patterns of gene expression. Here we determine how complex composite tissue structure affects measures of gene expression using RNA-seq.

**Results:**

We focus on two structures in the honey bee (the sting gland and digestive tract) both contained within one larger structure, the whole abdomen. For each of the three structures, we used RNA-seq to identify differentially expressed genes between two developmental stages, nurse bees and foragers. Based on RNA-seq for each structure-specific extraction, we found that RNA-seq with composite structures leads to many false negatives (genes strongly differentially expressed in particular structures which are not found to be differentially expressed within the composite structure). We also found a significant number of genes with one pattern of differential expression in the tissue-specific extraction, and the opposite in the composite extraction, suggesting multiple signals from such genes within the composite structure. We found these patterns for different classes of genes including transcription factors.

**Conclusions:**

Many RNA-seq studies currently use composite extractions, and even whole insect extractions, when tissue and structure specific extractions are possible. This is due to the logistical difficultly of micro-dissection and unawareness of the potential errors associated with composite extractions. The present study suggests that RNA-seq studies of composite structures are prone to false negatives and difficult to interpret positive signals for genes with variable patterns of local expression. In general, our results suggest that RNA-seq on large composite structures should be avoided unless it is possible to demonstrate that the effects shown here do not exist for the genes of interest.

## Background

RNA-seq is revolutionizing the study of gene expression. RNA-seq has been shown to be quantitatively accurate over a larger range of expression levels than previous methods, such as microarrays, while also being more effective at identifying genes that show low expression levels [1-7]. RNA-seq is also leading to major breakthroughs in the study of functional RNAs and gene regulation [7-10]. Studies of large scale patterns of expression of microRNAs have shown the fundamental roles these molecules play in regulating transcripts, while studies of long non-coding RNAs have revealed an unforeseen depth of functional roles for these genes in gene regulation and epigenetics [11-16].

While the technical and experimental logistics of how best to use RNA-seq are being addressed in a variety of contexts [17-20], one question that has received relatively little attention is the extent to which structure specific extractions are necessary for an accurate determination of gene expression. We use the terms “structure” and “organ” interchangeably, as we are referring to biological structures with distinct functions within a larger whole. In insects, for example, structures (organs) such as the fat body and segmental ganglia are contained within the abdominal body segment. We refer to large structures, such as the abdomen, with many internal structures as “composite structures”. Essentially, although efforts are under way to develop procedures for effective isolation of structures for RNA extraction (even particular cell types within complex tissue) [21-25], there is little experimental support for composite structure extractions actually being difficult to interpret or prone to error. This has led to widespread use of composite structures in RNA-seq studies of small organisms such as insects [26-31].

Although many studies are using composite extractions, there are potential problems with this approach. First, if genes are expressed in many different structures within the composite structure, then signals of gene expression from the different organs may interfere with one another. For example, if the structure of interest is small relative to the size of the rest of the structure (a gland within a whole larva, for example), then a strong difference in gene expression within the gland may be washed out by different patterns of expression elsewhere. Second, incomplete homogenization of the composite structure during extraction may lead to little tissue from particular structures actually being extracted. Hence, there are straightforward reasons to suspect that RNA-seq from composite-structures such as body segments may cause false negatives and difficult to interpret patterns of differential expression.

Here we explore the necessity of using structure (organ) specific extractions for RNA-seq using three honey bee structures. We focus on two structures, the sting gland and the digestive tract, that occur within one body segment, the whole abdomen. The sting gland is a relatively small structure within the larger composite structure that can be predicted to expresses a large number of specialized genes. These include the many venom proteins that make up honey bee venom, along with the enzymatic machinery used to produce and modify these proteins [32,33]. Exploring patterns of differential gene expression in sting gland specific extractions and comparing them to patterns of expression in the whole abdomen extractions can address the question of how much error can be expected when a composite extraction is conducted but the focal tissue is a small specialized structure within it. The digestive tract is a large structure not thought to be highly specialized (though many digestive enzymes undoubtedly show tissue specific expression patterns). By comparing patterns of gene expression in digestive tract specific extractions to patterns in the whole abdomen we can explore how much error is to be expected when the focal structure is a sizeable portion of the composite structure. Both comparisons will shed light on whether contrasting patterns of gene expression in different structures and false negatives in general are a problem for RNA-seq using composite structures.

## Results and discussion

### Transcriptome characterizations based on structure-specific or composite extractions

**Figure 1:**
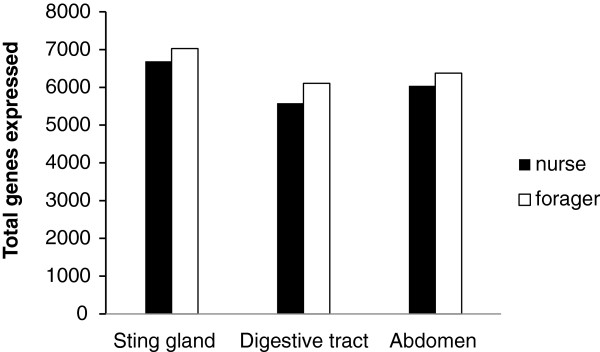
**Total transcriptome size of each structure in nurse and forager honey bees.** Genes with RPKM > 10.

The simplest potential problem with composite extractions is that genes that are expressed in small structures within the composite structure may be falsely determined to be not expressed in the composite structure. This may be a particularly pressing problem for genes with important functions that show low expression levels in small structures. We began our exploration of this issue by first determining the total number of genes expressed in each structure (Figure 1; genes and expression levels in Additional file 1: Table S1). The sting gland showed the largest transcriptome size, while the abdomen and digestive tract showed lower levels of expression. These numbers are for the same number of reads from each strucuture (12 million reads), so they presumably represent differences in how comprehensively the transcriptome was canvassed at this sequencing depth in conjunction with the complexity of the transcriptome. Hence, with respect to the sting gland versus the digestive tract, it is possible that the sting gland contains a higher number of expressed genes, or it is possible that the coverage level is sufficient to completely document expression in the sting gland, while it is insufficient to document all the genes expressed in the digestive tract. While this question is not resolvable with the current data set, the comparison of the sting gland transcriptome size to that of the whole abdomen transcriptome size is more straightforward. Here, since the sting gland is contained within the abdomen, the abdomen must have a larger transcriptome size. Hence, the fact that the sting gland was determined to have a larger transcriptome than the whole abdomen means that the whole abdomen has not been sequenced at sufficient depth to identify all the genes present within it. Hence, unless an RNA-seq study sequences at sufficient depth to perform an analysis proving that the number of genes found to be expressed is the total level (transcriptome size plateaus with increasing sequencing depth), there will always be concern that genes with low expression levels in particular tissues within the composite structure are missing from the data set.

**Figure 2:**
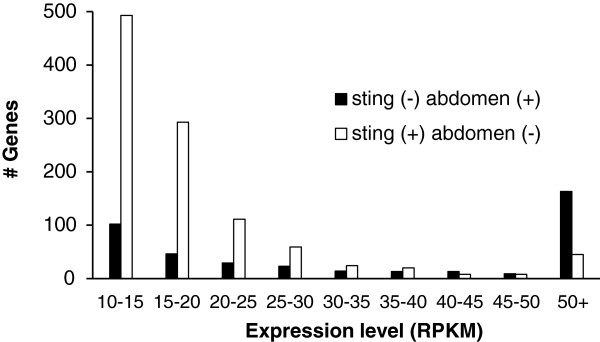
**Genes present or absent in the abdomen relative to sting gland transcriptome.** Number of genes expressed (at greater than 10 RPKM) in the sting gland missing in the abdomen, along with the number of genes missing in the sting gland that were present in the whole abdomen. (+) indicates the gene was present in a transcriptome, while a (-) indicates it was missing. Data are for genes present or absent in nurse transcriptomes.

**Figure 3:**
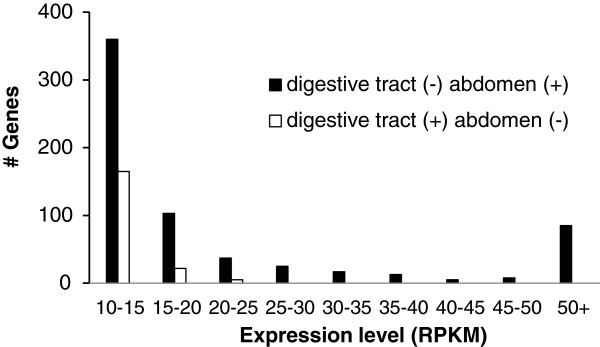
**Genes present or absent in the abdomen relative to digestive tract transcriptome.** Number of genes expressed in the digestive tract missing in the abdomen, along with the number of genes missing in the digestive tract that were present in the whole abdomen. (+) indicates the gene was present in a transcriptome, while a (-) indicates it was missing. Data are for genes present or absent in nurse transcriptomes. Only genes with RPKM > 10 included.

Figures 2 and 3 follow up on the results of Figure 1 by attempting to discover which of the genes present in the structure specific extractions are missing in the composite extractions and why. Figure 2 shows genes that are present in the sting gland transcriptome, but not present in the whole abdomen transcriptome and vice versa (genes and expression values in Additional file 2: Table S2). Analyses are for the nurse bee transcriptomes. Forager transcriptome comparisons showed the same patterns and are therefore omitted. For genes that were present in the sting gland, but not in the whole abdomen, most of them show low expression levels suggesting they were missing from the whole abdomen data set due to insufficient coverage. However, a small, but significant, number of genes showed relatively high expression in the sting gland (over 100 RPKM) and were nonetheless missing from the whole abdomen data set (Additional file 2: Table S2). For these genes, insufficient coverage as the cause for their being missing from the abdominal transcriptome is less parsimonious than the alternative possibility, which is that random error in the sampling from the whole abdomen caused some genes with high local expression to be missing from the composite structure transcriptome. This problem exists because the optimal amount of starting material for RNA extraction is much smaller than the size of many structures, such as the abdomen, yet the researcher wants a sample that is representative of the whole composite tissue. We used the common method for solving this problem, which is grinding in liquid nitrogen before taking a sample for RNA extraction [26,31]. Although grinding in liquid N_2_ generates a fine powder of tissue that is easily mixed, the sample may still be insufficiently homogenized to eliminate strong stochasticity in the amount of each sub structure that makes it into the extraction. This is because the size of the powdered grains of tissue can still be significant relative to the size of small glands. Our data suggests that grinding tissue to a powder in liquid N_2_ may be insufficient to ensure complete homogenization and random sampling from a large structure. A simple solution may be to extract RNA from a much larger amount of ground sample (essentially conduct multiple extractions from one sample) and then sample a subset of the pooled RNA for downstream analyses.

For genes that were present in the whole abdomen, but not present in the sting gland, the pattern was different than that for genes present in the sting gland but not the abdomen (Figure 2). Here the mode class of genes had high expression in the abdomen (not low expression as for genes present in the sting gland and missing from the abdomen). The genes present in the abdomen, but missing from the sting gland transcriptome, are therefore presumably highly expressed specialized genes not expressed in the sting gland. The sting gland itself, for example, has many venom proteins with high expression levels that presumably would not be expressed in other structures in the abdomen. Genes with equally specialized functions are likely found in other structures as well.

Figure 3 shows the results for the same type of analysis for the digestive tract and the whole abdomen (genes and expression values in Additional file 2: Table S2). The pattern here is quite different from that with respect to the sting gland versus the whole abdomen, presumably due to the sting gland being a tiny structure within the whole abdomen, and the digestive tract being quite a large structure. In this case, genes that were present in the digestive tract, but missing from the whole abdomen, are all genes with low expression levels (there are no highly expressed genes in the digestive tract missing from the abdomen). Hence, the issue of missing key genes may not be a problem for large focal structures within composite structures, as the composite extraction contained all the genes except those with very low expression in the focal tissue. This is true for transcriptome characterization, but as we will see in the next section does not hold for RNA-seq. The opposite comparison, genes that were found in the whole abdomen but missing from the digestive tract showed a pattern more like that found for the sting gland versus the abdomen comparison. In short, many genes with high expression levels in the abdomen were not found in the digestive tract presumably because they exhibit specialized functions elsewhere in the abdomen.

### Errors in diagnosing differentially expressed genes due to composite structure extraction

The major hypothesized problem associated with composite structure extraction and RNA-seq is contrasting gene expression patterns for the same genes in different structures interfering with a determination of differential expression. For example, when a researcher conducts an RNA-seq study on a whole body segment and finds that a gene is not differentially expressed, does it mean that it is not differentially expressed anywhere in the body segment or does it mean that there is no overall difference in expression level when summing the inputs from all the internal organs? Essentially, it is easy to imagine that a gene that is strongly differentially expressed in one organ may be determined to be non-differentially expressed in a much larger composite structure due to expression of the gene elsewhere washing out the signal from the small structure. Hence, there could be a strong false negative problem with composite extractions. Likewise, it is easy to imagine that a gene with contrasting differential expression patterns in different structures may give a strong signal of being differentially expressed in one direction that is representative of a strong signal from one structure washing out several opposite signals from other structures. Hence, although a determination of differential expression in a composite extraction is not a false positive, it can be difficult to interpret.

**Figure 4:**
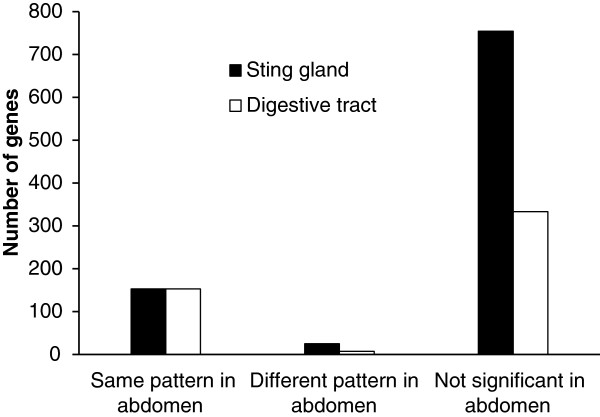
**Genes with the same or different pattern of expression in composite relative to tissue specific extractions.** Number of genes found to be differentially expressed between nurse and forager castes using RNA-seq for both the sting gland and digestive tract that were either found to be not differentially expressed in the corresponding whole abdomen tests, or were differentially expressed but in the opposite direction. Results based on NOISeq analysis.

Figure 4 explores these potential problems for identifying differentially expressed genes in the sting gland and digestive tract. We conducted three RNA-seq analyses: nurse bee sting glands versus forager sting glands, nurse bee digestive tracts versus forager digestive tracts, and nurse bee abdomens versus forager abdomens. We used three RNA-seq software packages (NOISeq, EdgeR, and DESeq) with two biological replicates for each sample and a total of 12 million quality controlled reads for each sample. In total, we found (NOISeq: 932, DESeq: 781, EdgeR: 1279) differentially expressed genes in the sting gland between nurses and foragers, (NOISeq: 493, DESeq: 333, EdgeR: 770) in the digestive tract, and (NOISeq: 425, DESeq: 313, EdgeR: 637) in the whole abdomen. From here on we present the NOISeq results in the main text and the results for the other two packages in the supplemental information (Additional file 3: Figure S1), as all analyses led to the same basic conclusions. For each gene differentially expressed in the sting gland or the digestive tract, we determined whether that gene was significantly differentially expressed in the whole abdomen, and if so, in what direction (the same or different from the focal tissue). In other words, if the gene was found to be expressed at higher levels in nurses relative to foragers in the sting gland, was it also found to be expressed at higher rates in nurses in the whole abdomen? For the sting gland, 754 out of 932 differentially expressed genes in the sting gland were found to be not differentially expressed in the whole abdomen (Figure 4). For the digestive tract, 340 out of 493 genes differentially expressed in the digestive tract comparison were not differentially expressed in the whole abdomen comparison (Figure 4). Gene names, expression values, and p values from each analysis are in supplemental Additional file 4: Table S3. Nearly identical patterns were found for the other software packages (Additional file 3: Figure S1), so the effects are not caused by the statistical algorithm used to identify differentially expressed genes.

**Figure 5:**
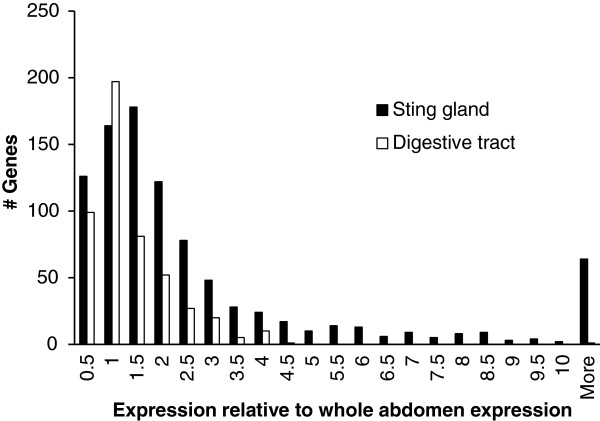
**Distribution of ratios of the expression of each gene in both the sting gland and the digestive tract relative to the whole abdomen.** A value of 1 means that the gene’s RPKM value was equal in the tissue specific and the composite extraction, while a value above 1 means that the gene was expressed at a higher rate in the tissue specific extraction and showed dilution in the composite extraction. Genes showing dilution are candidates for specialized function.

We next shed light on the patterns found in Figure 4 by plotting the ratio of expression levels for genes in both focal tissues relative to that in the composite tissue (using the NOISeq RPKM analysis). A gene with a ratio of 1, for example, would be expressed at the same level in both the focal and composite tissue, implying that it is not specialized (expressed at a higher rate in the focal tissue). In other words, we are comparing the RPKM value for each gene in the sting gland or digestive tract with that for the same gene in the abdomen. We focus on the nurse libraries. The forager libraries showed the same pattern and are therefore omitted. Figure 5 shows that the sting gland has many more genes with higher levels of expression relative to that in the abdomen (ratios above 1) compared to the digestive tract (Sting gland: 642 out of 932 (68.9%), Digestive tract 197 out of 493 (40.0%); Fisher’s exact test: *p* < 0.001). Hence, more genes show a dilution of expression level in the whole abdomen relative to the sting gland, than for the abdomen relative to the digestive tract. This could explain why a higher percentage of genes that were significantly differentially expressed in the sting gland were not significant in the whole abdomen, relative to the same comparison between the digestive tract and the abdomen (Sting gland: 754 out of 932 (80.9%), Digestive tract: 340 out of 493 (69.0%); Fisher’s exact test: *p* < 0.001). Overall, our data supports the notion that false negatives are a serious problem in RNA-seq with composite structures, such as body segments, and may be particularly pressing for genes with specialized functions in small structures, such as glands.

With respect to genes potentially showing the opposite pattern of differential expression in a composite structure relative to a smaller structure within it, 25 such genes were identified in the sting gland, and 7 in the digestive tract (Figure 4, very similar results for DESeg and EdgeR shown in Additional file 3: Figure S1). These contrasting signals of differential expression between tissue specific and composite tissue analyses suggests there may be a rich pattern of variation of function across tissues for such genes. This would make interpreting expression patterns for those genes quite difficult in composite extractions. This result suggests again that composite tissue extractions can be difficult to interpret and might best be avoided whenever possible.

**Figure 6:**
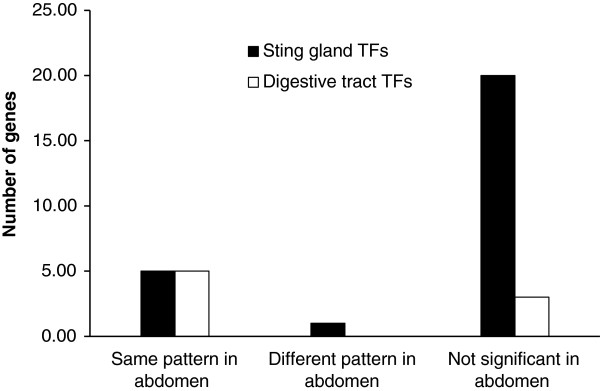
**Transcription factors with the same or different pattern of expression in composite relative to tissue specific extractions.** Number of transcription factors found to be differentially expressed between nurse and forager castes using RNA-seq in both the sting gland and digestive tract that were either found to be not differentially expressed in the corresponding whole abdomen tests, or were differentially expressed but in the opposite direction. Results based on NOISeq analysis.

Finally, transcription factors are a class of genes that are well known to be used repeatedly in different structures in contrasting manners. They are therefore a class of gene for which composite extractions could be an acute problem. Figure 6 repeats the analysis conducted in Figure 4 for just the differentially expressed transcription factors (results for EdgeR and DESeq are in Additional file 5: Figure S2). In total, there were 26 differentially expressed transcription factors in the sting gland nurse bee to forager comparison, 8 in the nurse bee to forager digestive tract comparison, and 9 in the nurse to forager abdomen comparison (Additional file 6: Table S4). Most differentially expressed transcription factors in the sting gland were not differentially expressed in the abdomen (20 out of 26 were not significant), meaning that the problem of false negatives is also true for transcription factors. One transcription factor (Dorsal) showed the opposite pattern of expression in the whole abdomen relative to the sting gland. The numbers for the digestive tract (only 8 differentially expressed transcription factors) mean the sample size is too small for any comparisons between structures, but descriptively, most transcription factors differentially expressed in the digestive tract were also differentially expressed in the abdomen (5 out of 8) and none were in the opposite direction. In summary, only 1 transcription factor was found to show the opposite pattern of expression in the composite structure relative to the organ specific analysis, but false negatives were common. Given the role that transcription factors play in controlling the expression of many other genes, such errors may be more significant than for other classes of genes.

## Conclusions

Making sequencing libraries from composite structures, such as body segments, and even whole insects is commonplace in RNA-seq studies [26-31]. Our results suggest that this practice can lead to false negatives for genes that show even strong patterns of differential expression in particular structures (organs). For genes that show complex patterns of variable expression in different structures across the organism, it is further likely that composite extractions are of little utility other than for identifying genes as candidates of interest. This is because it is difficult to infer the direction of differential expression in composite structures when more than one pattern may be present. Finally, our study focused on the abdomen, which is a relatively simple composite structure in comparison to a whole larval insect. It is likely that the types of errors documented here associated with identifying differentially expressed genes will be magnified in studies using whole organisms. Hence, given that structure specific extractions are usually possible for even very small structures [34-37], they should be conducted whenever possible.

## Methods

### Colonies and collection of bees

Honey bees were kept according to standard beekeeping practices at the bee biology facility at UC Davis. Two full size colonies were used in the study. All colonies were healthy and had been undisturbed for at least a month prior to collection of bees. Nurses were collected by identifying bees with their head and thorax completely in an open brood cell for at least three seconds [38,39]. A further check to ensure that bees identified in this way were nurses was to check the developmental state of the Hypopharyngeal Glands (HP Glands) of each nurse at the start of each dissection. Nurses have large HP glands, as this gland produces the brood food fed by nurses to larva [40,41]. Foragers, in contrast, have smaller, often yellowish, HP glands, as this gland produces digestive enzymes, not brood food, in bees in the foraging caste [42]. Only bees with large white HP glands were kept as nurses. Foragers were collected as they entered the nest. Only foragers with pollen loads were used in the study. Immediately after collection, bees of both castes were placed into 50 ml centrifuge tubes and placed in the -80°C freezer.

### Dissection, extractions, and sequencing

Additional file 7: Figure S3 shows the dry mass of each structure used in the study for comparative purposes. Dissections and RNA extractions were carried out one after the other to minimize degradation of RNA during dissection. Dissections were conducted by removing individual bees from the freezer and placing them into a petri dish with 50% ethanol under a dissection scope. As soon as the bee thawed, the dissection began. Dissections were completed within 3-5 minutes. For the sting gland, which is designed to detach from the adult bee when it stings, the process of dissection is simple. The stinger is grasped with forceps and pulled. The entire sting gland with associated venom sac pulls away from the body. Thirty stingers were pooled for each biological sample. All individuals pooled into one sample were from the same colony. Hence, 30 individuals from colony 1 and thirty individuals from colony 2 were used in the study. As each stinger was removed it was immediately homogenized in Trizol. This was repeated for each subsequent dissection. After all 30 stingers were dissected and homogenized, total RNA was extracted according to the manufacturer’s recommendations.

For the digestive tract a similar procedure was used with a few modifications. First the digestive tract is a large structure, so only 3 could be extracted per 1.5 ml microtube. Hence, 10 tubes total were used (3 individual bee digestive tracts per tube). After all 30 individual samples were homogenized, the samples were pooled in one 50 ml centrifuge tube and vortexed. Then one sample of 1 ml was taken from the total for extraction in a 1.5 ml tube. For the abdomen, which is too large for complete extraction in one microtube, 30 bee abdomens were first ground in liquid N_2_ using a mortar and pestle. 75 mg of the resulting fine powder was then taken for RNA extraction with Trizol.

RNA quality was checked with the Bioanalyzer 2100 and libraries were made using Illumina’s TruSeq v2 kit according to the manufacturer’s recommendations. 100 bp paired end sequencing was then performed on the HiSeq 2000 machine. The raw fastq files from this study are available at the NCBI SRA archive (SRP020361).

### Quality control and RNA-seq analyses

Low quality bases and adapter contamination were removed with the fastx toolkit and the cutadapt software packages [43]. Tophat (v2.04) was used for aligning reads to the *Apis mellifera* genome [44] (v4, the most recent officially published version). HTSeq was used for quantifying the number of reads mapping to each gene. NOISEQ, EdgeR and DESeq were used to determine differential expression [19,45,46]. For NOISeq, RPKM normalization was used along with a 0.8 p cutoff (the recommended cut-off level). For EdgeR and DESeq, an adjusted p value (FDR) < 0.05 was used to call differentially expressed genes. All analyses made use of 2 biological samples and 12 million quality controlled paired end reads. Expression levels within biological replicates for the same tissue were highly correlated (mean: 98.3%, range 97.1% -99.6%).

### Identification of transcription factors

All *Drosophila melanogaster* genes with the GO term “sequence specific DNA binding” were downloaded from flybase and blasted against all genes in the official gene set of *Apis mellifera*. Genes with a hit (e < 10^-20^) to one of the *Drosophila* transcription factors that had a functional domain involved in DNA biding were kept. Overall, 462 genes passed this filter (Additional file 8: Table S5). While the resulting list is not exhaustive, in that there are surely many more *Apis* transcription factors, it is a large sample of transcription factors that should be broadly representative of this class of genes.

## Competing interests

The authors declare that they have no competing interests.

## Authors’ contributions

JA made the sequencing libraries, did quality control of the RNA and libraries, and revised the manuscript. DCP made the sequencing libraries, did quality control of the RNA and libraries, and revised the manuscript. BRJ collected the bees, extracted the RNA, designed the experiment, performed the bioinformatics, and wrote the manuscript. All authors approved the final submission.

## Supplementary Material

Additional file 1: Table S1 All genes found to be expressed in each tissue along with expression levels (RPKM). Click here for file

Additional file 2: Table S2 List of genes found to be expressed in the sting gland or digestive tract, but missing from the abdomen, or expressed in the abdomen, but missing from either the sting gland or digestive tract. Expression levels are in RPKM. Click here for file

Additional file 3: Figure S1 Same analyses shown in Figure 4 in the main text, but using **(A)** the DESeq R software package, and **(B)** EdgeR software package. Click here for file

Additional file 4: Table S3 List of genes found to be differentially expressed in either the sting gland or digestive tract between nurses and foragers, and their pattern of expression (significant or non-significant, and in the same direction or the opposite direction) in the whole abdomen RNA-Seq comparison between nurses and foragers. Click here for file

Additional file 5: Figure S2 Same analyses shown in Figure 6 in the main text, but using **(A)** the DESeq R software package, and **(B)** EdgeR software package. Click here for file

Additional file 6: Table S4 List of transcription factors found to be differentially expressed in either the sting gland or digestive tract between nurses and foragers, and their pattern of expression (significant or non-significant, and in the same direction or the opposite direction) in the whole abdomen RNA-Seq comparison between nurses and foragers. Click here for file

Additional file 7: Figure S3 Dry mass of 30 dissected sting glands, digestive tracts, and abdomens. Click here for file

Additional file 8: Table S5 Transcription factors identified in the honey bee genome. Click here for file
