# Supplementary figures and images for: The importance of tissue specificity for RNA-seq: highlighting the errors of composite structure extractions
Source: BMC Genomics. 2013 Aug 28;14:586. doi: 10.1186/1471-2164-14-586 (PMC3765781; doi:10.1186/1471-2164-14-586)

A.

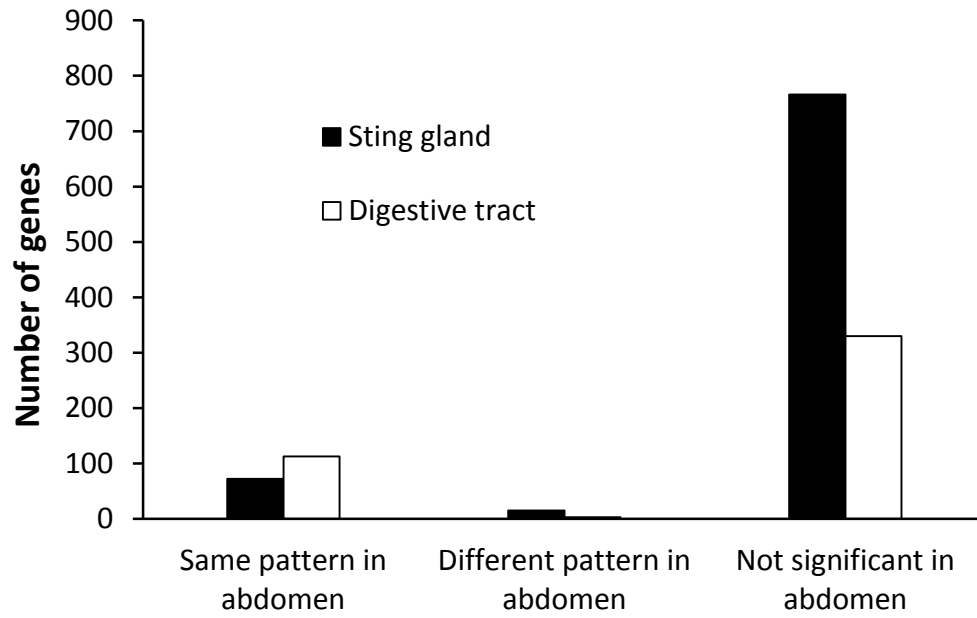

B.

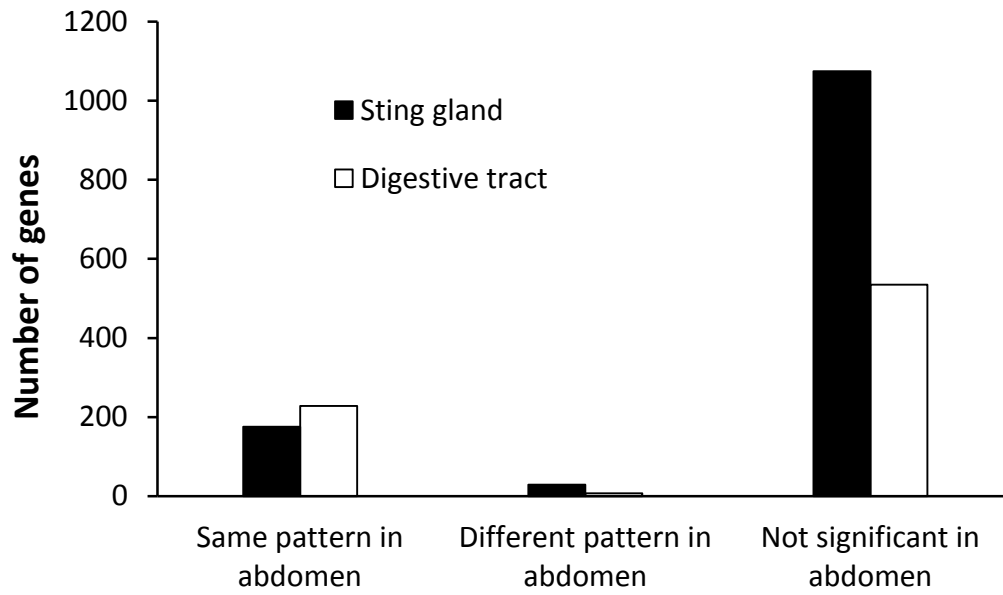

Supplement: Additional file 3: Figure S1 — Same analyses shown in Figure 4 in the main text, but using (A) the DESeq R software package, and (B) EdgeR software package. [file 1471-2164-14-586-S3.pdf]

A.

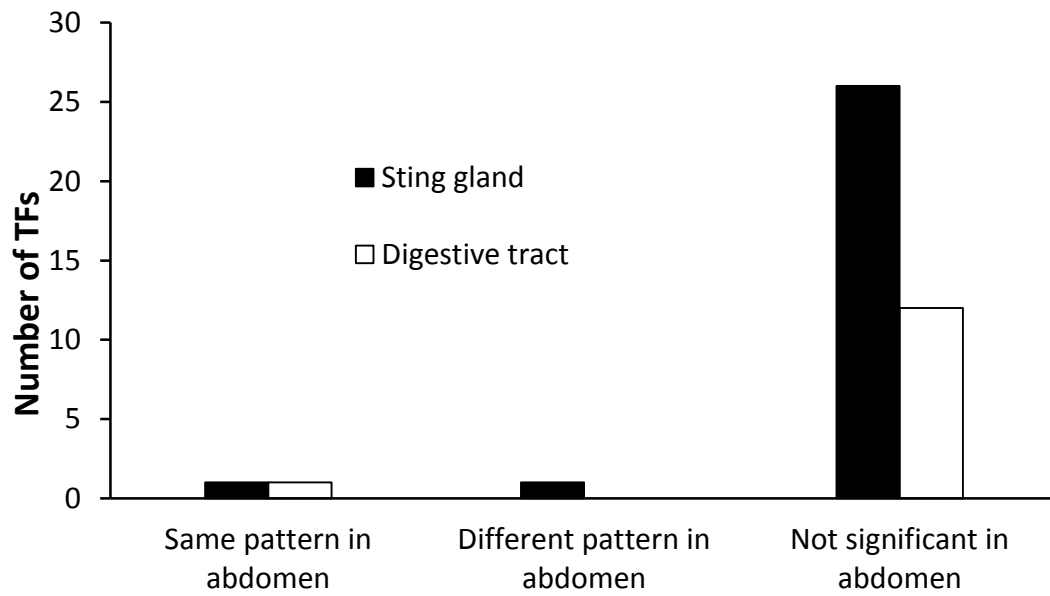

B.

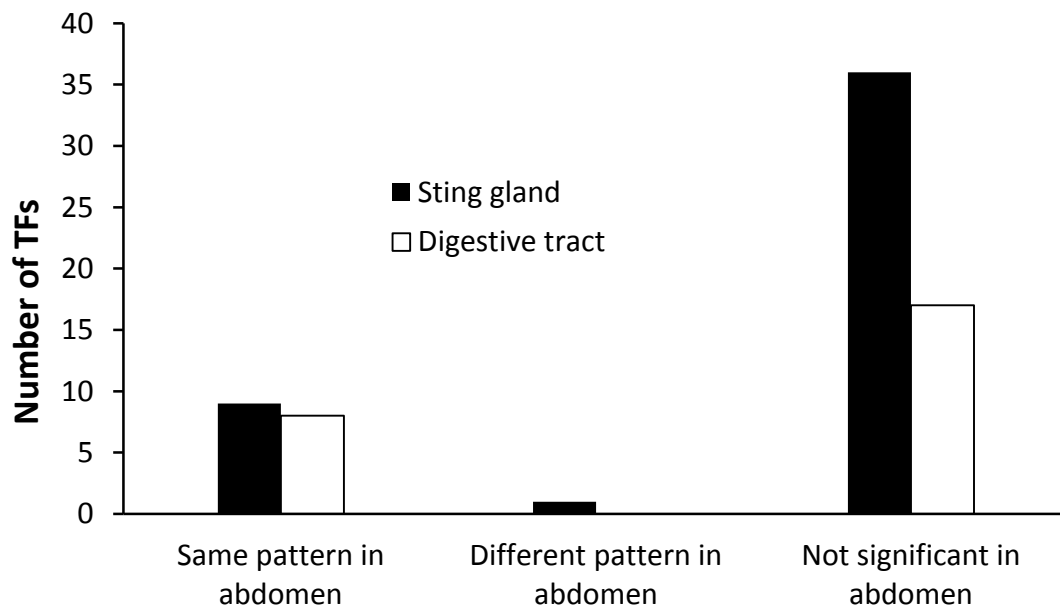

Figure S2.

Supplement: Additional file 5: Figure S2 — Same analyses shown in Figure 6 in the main text, but using (A) the DESeq R software package, and (B) EdgeR software package. [file 1471-2164-14-586-S5.pdf]

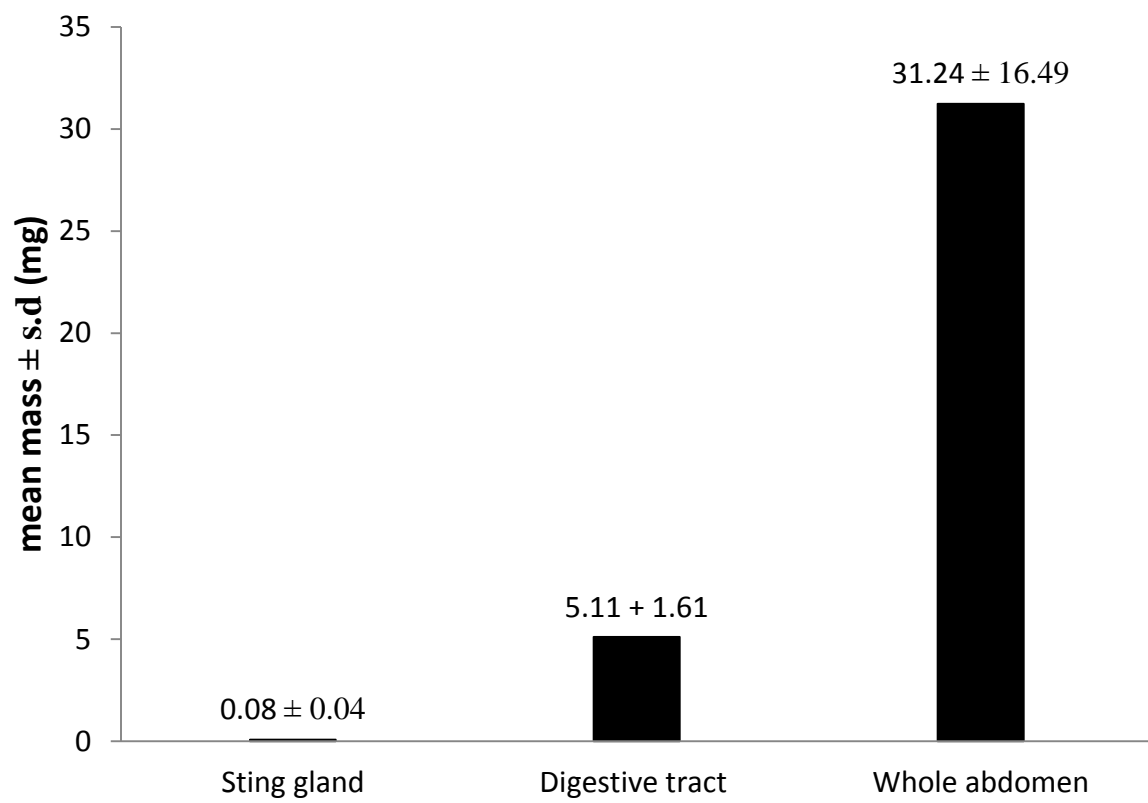

Supplement: Additional file 7: Figure S3 — Dry mass of 30 dissected sting glands, digestive tracts, and abdomens. [file 1471-2164-14-586-S7.pdf]
